# Mitophagy plays a “double-edged sword” role in the radiosensitivity of cancer cells

**DOI:** 10.1007/s00432-023-05515-2

**Published:** 2024-01-18

**Authors:** Qian Wang, Chengxin Liu

**Affiliations:** 1 The First School of Clinical Medicine, Lanzhou University, Lanzhou, 730030 Gansu China; 2 Shandong Academy of Medical Sciences, Shandong Cancer Hospital and Institute, Shandong First Medical University, Jinan, 250117 Shandong China

**Keywords:** Mitophagy, Radiosensitivity, Cancer, Ionizing radiation

## Abstract

Mitochondria are organelles with double-membrane structure of inner and outer membrane, which provides main energy support for cell growth and metabolism. Reactive oxygen species (ROS) mainly comes from mitochondrial and can cause irreversible damage to cells under oxidative stress. Thus, mitochondrial homeostasis is the basis for maintaining the normal physiological function of cells and mitophagy plays a pivotal role in the maintenance of mitochondrial homeostasis. At present, to enhance the sensitivity of cancer cells to radiotherapy and chemotherapy by regulating mitochondria has increasingly become a hot spot of cancer therapy. It is particularly important to study the effect of ionizing radiation (IR) on mitochondria and the role of mitophagy in the radiosensitivity of cancer cells. Most of the existing reviews have focused on mitophagy-related molecules or pathways and the impact of mitophagy on diseases. In this review, we mainly focus on discussing the relationship between mitophagy and radiosensitivity of cancer cells around mitochondria and IR.

## Introduction

**Fig. 1:**
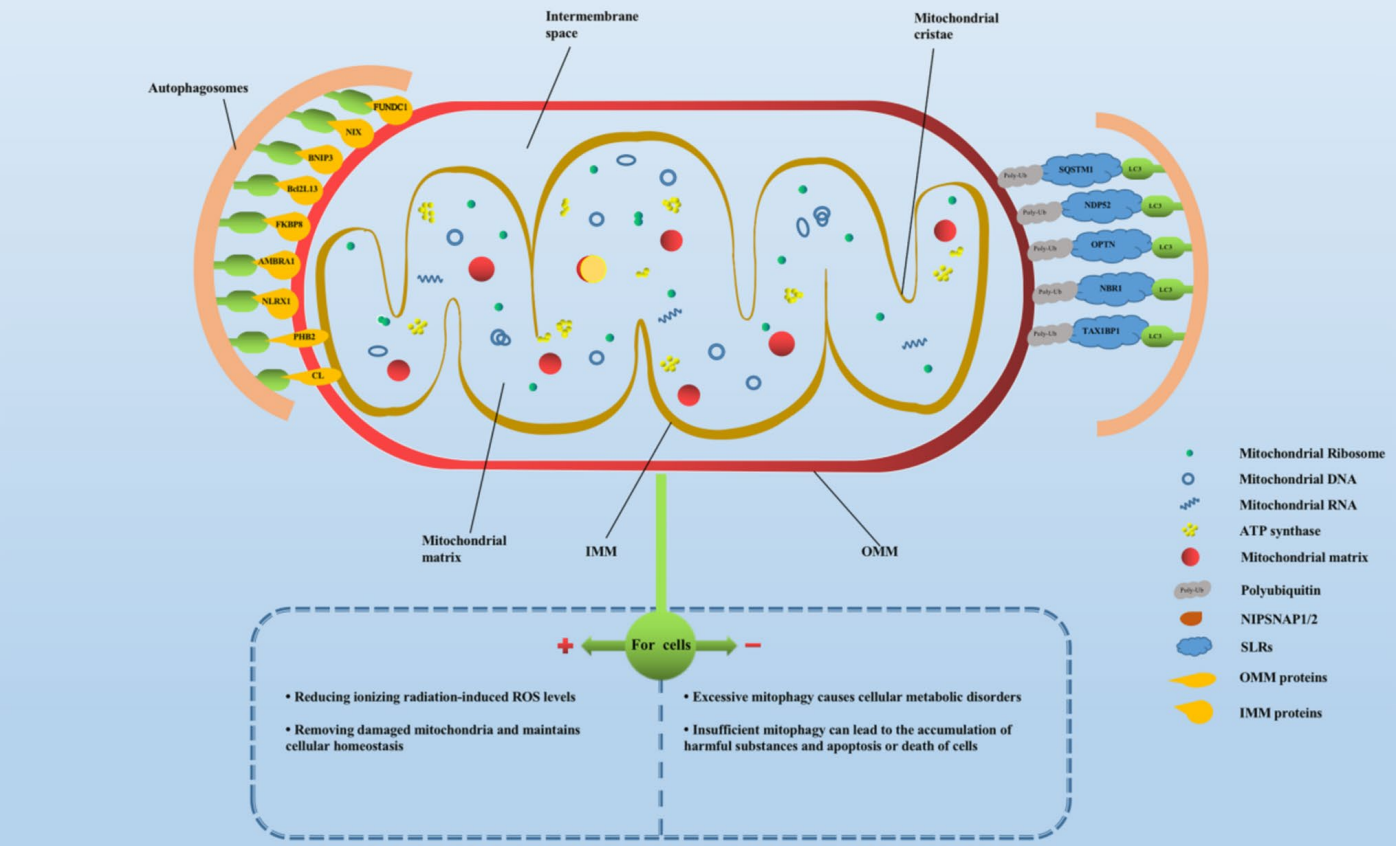
Schematic diagram of mitochondrial structure and the distribution of mitophagy-related molecular receptors on mitochondria. *SLRs* SQSTM1-like receptors; *OMM* outer mitochondrial membrane; *IMM* inner mitochondrial membrane; *ROS* reactive oxygen species

Mitochondria are double-membrane organelles present in most eukaryotic cells, and mitochondria are the main sites of intracellular oxidative phosphorylation and formation of adenosine triphosphate (ATP) (Herst et al. 2017; Pfanner et al. 2019). It can be divided into four functional areas: outer mitochondrial membrane (OMM), membrane space, inner mitochondrial membrane (IMM) and matrix from the outside to the inside. As shown in Fig. 1, the IMM possesses a several-fold larger surface than the OMM, resulting in an invagination of so-called cristae membranes that harbor the oxidative phosphorylation system, including the respiratory complexes I–IV and the F_1_F_O_-ATP synthase for ATP. Therefore, major metabolic pathways of mitochondria concern the energy metabolism, such as the tricarboxylic acid cycle (TCA) also known as citric acid cycle or Krebs cycle, and the metabolism of amino acids, lipids and nucleotides (Pfanner et al. 2019). Besides, mitochondria are involved in processes, such as heme biosynthesis, calcium buffering, apoptosis, and innate immune surveillance (Franco-Iborra et al. 2018; Ni et al. 2015). The metabolism of mitochondria mentioned above produces a large amount of ROS, includes the superoxide anion (O_2_^−^), hydrogen peroxide (H_2_O_2_), hydroxyl radical (·OH), and singlet oxygen (^1^O_2_) (Han et al. 2018).

Recent studies showed that ROS levels correlate with the radiosensitivity of cells. There are two major sources for ROS production in cells: mitochondria, which generate ROS as a by-product of respiration; NADPH oxidase (NOX), which actively produces superoxide across the membranes of neutrophils and phagosomes (Li et al. 2015). On the other hand, intracellular ROS remains at a relatively low level and is precisely controlled by endogenous free radical scavengers, such as superoxide dismutase, catalase, and the glutathione peroxidase and thioredoxin reductase systems (Venardos et al. 2007; Finkel and Holbrook 2000). Antioxidants capable of scavenging excessive ROS may help maintain oxidative homeostasis and prevent related damages. Various factors, such as toxins, oxidants and IR, can increase ROS level sharply and make the anti-oxidation defense systems in a weak position, leading to disruption of the balance between generation and elimination of ROS and consequently progression of diseases and even eventual death (Dalle-Donne et al. 2001; Yu et al. 2006; Motoori et al. 2001; Ayaka et al. 2012).

Mitochondrial homeostasis is particularly important for ROS level and maintained by an intricate balance between fission, fusion, mitochondrial biogenesis, and mitophagy (Chan 2012; Hoitzing et al. 2015). Mitophagy as a kind of selective autophagy is one of the main pathways for mitochondrial quality and quantity control. The word autophagy is derived from the Greek roots “auto” (self) and “phagy” (eating) (Li et al. 2020). As the name implies, mitophagy is an intracellular degradation pathway in eukaryotes, which depends on lysosomes, resulting in some damaged or excess aging mitochondria being swallowed and degraded. Previous studies have shown that ROS is closely related to autophagy (Yang et al. 2008; Pyo et al. 2008; Dadakhujaev et al. 2008; Chen et al. 2008a, b). And mitophagy also can be further stimulated by oxidative stress. Upon stress or injury, mitophagy prevents the accumulation of damaged mitochondria and the increased level of ROS leading to oxidative stress and cell death (Ashrafi and Schwarz 2013).

In summary, we hold the opinion that there is also a relationship between mitophagy and the radiosensitivity of cells, but there is no related review of this issues at present. Next, we will discuss the effects of IR on cancer cells and mitochondria, and the effects of mitophagy on the radiosensitivity of cancer cells.

## Effects of IR on cancer cells

### ROS and DNA

The killing effects of IR on cells are divided into direct and indirect effects. IR could cause irreparable single-strand or double-strand breaks of DNA, resulting in cell death, which belong to the direct effects of IR. Indirect effects refer to the reaction of IR with water molecules in cancer cells to form oxygen free radicals, causing oxidative stress, which is more damaging to cells. Among them, oxidative stress is larger and more serious to cells. Oxidative stress is defined as the imbalance between oxidants-ROS, Reactive Nitrogen Species (RNS) and antioxidants. Under oxidative stress, excessive accumulation of ROS could further aggravate oxidative damage using mitochondria as primary targets and destroy cellular proteins, lipids and DNA, resulting in fatal cell damage, which in turn involves a variety of pathology, such as aging, cancer, metabolic syndrome, neurodegenerative diseases, cardiovascular disease, diabetes, and so on (Ichimiya et al. 2020; Rodolfo et al. 2018; Drake et al. 2017).

### Radiosensitivity

The radiosensitivity of tumor cells is related to many factors, such as type of tumor cells, presence of cancer stem cells, type of radiation, and tumor microenvironment. More and more studies have shown that mitochondria also play an important role in the radiosensitivity of cells, energy metabolism, mitochondrial apoptosis, mitophagy, regulation of redox homeostasis, and calcium influx all play an indispensable role in this process (Lynam-Lennon et al. 2014; Yuehua et al. 2018; Yang et al. 2021). Mitochondria are the main source of energy for cell growth and proliferation. Various external stimuli lead to mitochondrial damage and loss of oxidative phosphorylation function, which will affect cell metabolic activities and eventually lead to cell apoptosis or death. This is also the main mechanism of action of many chemotherapy drugs at present. Therefore, mitochondria are increasingly becoming an important target for studying the effect of enhancing tumor killing.

## Effects of IR on mitochondria

### Elevated ROS levels

As mentioned above, ROS is a key medium of mitochondrial damage induced by IR, and one of the main sources of ROS is the respiratory chain of IMM (Addabbo et al. 2009; Zorov et al. 2014; Turrens 2003). ROS production of mitochondria will increase in cells after being irradiated. Hosoki et al. revealed that the cellular level of ROS increased in HeLa S3 cells during post-irradiation (Ayaka et al. 2012). Motoori et al. used di-hydro-rhodamine 123 to detect mitochondrial ROS and radiation incubation using flow cytometry with a fluorescent probe showed a radiation-induced elevation in mitochondrial ROS in human hepatoma cells (Motoori et al. 2001). Zhang et al. showed that small airway epithelial exposed to 10α particles through the cytoplasm resulted in an increase in fluorescence intensity of superoxide production at 2 h post irradiation at a level that was three times that of control (Zhang et al. 2013).

### Mitochondrial membrane potential (ΔΨm) depolarization

The IMM is distributed with many proton pumps, whose function is to pump intra-matricial protons (H^+^) into the outer side of the inner membrane, thus forming ΔΨm across the inner membrane to maintain the normal function of mitochondria. When mitochondria are exposed to IR, it can lead to obstacles in the electron transport process of the respiratory chain and affect the formation of H^+^ transmembrane gradient, which will lead to a decrease in the ΔΨm of the original external positive and internal negative, that is, ΔΨm depolarization. Mitochondrial depolarization signals cause autophagy-related proteins, such as PINK, to stabilize the OMM and accumulate, initiating the process of mitophagy (Ashrafi and Schwarz 2013).

### Mitochondrial DNA (mtDNA) damage

Human mtDNA is a circular double-stranded molecule with a full length of 16,569 bp and it can be divided into heavy chain and light chain.

MtDNA accounts for only 1–2% of the total human DNA, it has no intron and contains 37 genes encoding 13 polypeptides, 2 ribosomal RNAs (rRNAs), and 22 transfer RNAs (tRNAs) (Mishra and Chan 2014; Anderson et al. 1981). There is also the only two non-coding regions, namely D-loop and the replication start point of the light chain, the former is the main regulatory region of mtDNA replication and transcription. The part of nuclear DNA that is involved in protein coding accounts for only 1% of the total human DNA, so scholars generally believe that damage of mtDNA is more likely to lead to disease than nuclear DNA (Birney et al. 2007). Specifically, it is as follows: (1) mtDNA is bare and lacks histone protection; (2) The high fat/DNA value in mitochondria makes lipophilic carcinogens preferentially aggregate on mtDNA; (3) mtDNA is in a state of continuous synthesis throughout the cell cycle, which is susceptible to external interference and poor stability; (4) The high concentration of oxygen in mitochondria is easy to produce oxygen free radicals and hydrogen peroxide, and due to the scarce synthesize glutathione, mtDNA is susceptible to oxidative damage; (5) High frequency of replication mismatch; (6) With the not-so-perfect mechanism for repairing DNA damage (West and Shadel 2017; Yoshida et al. 2012; Clayton et al. 1974; Robert et al. 1975; Croteau et al. 1999; Larsen et al. 2005; Heddi et al. 1999; Penta et al. 2001). To sum up, the damage of mtDNA will be more obvious when cells are exposed to IR. Many scholars also have confirmed that ROS can cause more extensive and lasting damage to mtDNA, and mtDNA depletion can also in turn affect cellular oxidative stress (Yakes and Houten 1997; Azzam et al. 2012; Kim et al. 2008; Zhou et al. 2011; Kobashigawa et al. 2011; Garza-Lombó et al. 2020).

### Mitochondria swelling

Mild mitochondrial swelling under physiologic conditions regulates metabolism and function of mitochondria, whereas excessive swelling causes mitochondrial dysfunction (Makarov et al. 2020). The abnormal accumulation of ROS could upregulate inositol triphosphate, resulting in the opening of inositol triphosphate receptor-calcium ion channels on the endoplasmic reticulum membranes, calcium ions are released from the endoplasmic reticulum. The high level of calcium ion/calmodulin kinase (CaMK) in the cytoplasm can open the L-type voltage-gated calcium ion channel of the plasma membrane and the inflow of extracellular calcium ions, which further induces the opening of the mitochondrial bilayer membrane permeable pores, making the mitochondria swell and rupture (Orrenius et al. 2015).

## Mitophagy

As we can see from the above, ROS plays a particularly important role in the effects of IR on cells and mitochondria, and mitochondria are the main sites to product ROS. In addition to ROS, other IR-induced alterations in mitochondria can also initiate a series process, such as mitochondrial elimination, fission and fusion, and mitochondrial biogenesis, to maintain mitochondria homeostasis. Whole mitochondrial elimination is accomplished by a selective form of autophagy: mitophagy. The activation of mitophagy is closely related to the redox of cells, such as hypoxic environment and IR, can put cells under oxidative stress and induce the occurrence of mitophagy. Next, we made a review of mitophagy-related molecular receptors and pathways.

## Mitophagy-related molecular receptors (shown in Fig. 1)

Various metabolic activities in cells are transmitted by signaling pathways and molecules, and in recent years, great progress has been made in the study of mitophagy mechanism. Microtubule associated protein 1 light chain 3 (LC3) is the most used autophagy marker. LC3 gene is the mammalian homologous gene of autophagy-related protein8 (ATG8), which encodes three proteins: LC3A, LC3B and LC3C. During autophagy, LC3B cleaves to soluble protein LC3B-I, which combines with phosphatidylethanolamine to form LC3B-II. LC3B-II can accumulate in large quantities on the surface of neonatal autophagosomes, which is one of the reliable markers of autophagy (Zhihong et al. 2013). When mitophagy starts, LC3B-II binds to the proteins on mitochondria, causing autophagosomes to wrap damaged mitochondria to form mitochondrial autophagosomes, which is a key step in the occurrence of mitophagy. There are a variety of mitophagy receptors on the mitochondrial membrane, which are listed in Table 1. When cells are irradiated to cause mitochondrial injury or depolarization, such as the most common oxidative stress, changes in the mitochondria themselves will stimulate the changes of autophagic proteins, initiating mitophagy.

**Table 1:** Mitophagy-related molecular receptors

Type	Mitophagy receptor	Location	Is it ubiquitin-dependent?	Other features	References
SLRs	SQSTM1/p62	OMM	Yes	Coordinate stem cell differentiation, quench inflammation and tumor stroma recovery	Sabbieti et al. (2022)
	CALCOCO2/NDP52	OMM	Yes	Xenophagy and immune response	Leymarie et al. (2017), Michael et al. (2015), Morriswood et al. (2007)
	OPTN	OMM	Yes	Tumor suppression	Qiu et al. (2022)
	NBR1	OMM	Yes	Cancer metastasis and immune evasion	Kirkin (2009), Rasmussen et al. (2022)
	TAX1BP1	OMM	Yes	Negative regulation of cell growth and apoptosis	Kim et al. (2008)
Interact with LC3 directly	PHB2	IMM	No	Maintain the stability of mitochondrial genome	Yan et al. (2020)
	CL	IMM	No	Regulate cellular apoptosis	McMillin and Dowhan (2002)
	PINK1	OMM	No	–	**–**
	Parkin	OMM	No	–	**–**
	FUNDC1	OMM	No	Mitochondrial quality control	Chen et al. (2016a, b)
	NIX/BNIP3L	OMM	No	Regulate cellular apoptosis	Lampert et al. (2019)
	BNIP3	OMM	No	Regulate cellular apoptosis	Zhang and Ney (2009)
	Bcl2L13	OMM	No	regulate cellular apoptosis	Meng et al. (2021)
	FKBP8	OMM	No	Regulate cellular apoptosis	Misaka et al. (2018)
	AMBRA1	OMM	No	Tumor inhibition	Di Rita et al. (2018), Liu et al. (2017), Sun et al. (2018) Sun et al. (2019)
	NLRX1	OMM	No	Regulate cellular death, apoptosis and inflammation	Imbeault et al. (2014), Soares et al. (2014)

*SLRs* SQSTM1-like receptors; *OMM* outer mitochondrial membrane; *IMM* inner mitochondrial membrane

### SQSTM1-like receptors

Mitochondrial receptor proteins act as junctions between autophagosomes and mitochondria, one of which is called SQSTM1-like receptors (SLRs). In addition to interacting with LC3, these proteins have domains that bind to ubiquitin, through which autophagosomes and mitochondria are connected. Currently, SLRs include SQSTM1, CALCOCO2/NDP52, OPTN, NBR1, and CALCOCO3/TAX1BP1.

SQSTM1/p62 assists selective macro-autophagy and acts as a molecular sentinel in the auto-phagosome membrane for the recognition, sequestration, and degradation of intracellular wastes. In addition, p62 is defined as the odd-jobber protein, able to orchestrate autophagy, coordinate stem cell differentiation, quench inflammation and actively participate in tumor stroma recovery through immune cells recruitment and anti-metastatic activity (Sabbieti et al. 2022).

CALCOCO family proteins are the newly found selective autophagy receptors, which include CALCOCO1, CALCOCO2/NDP52, and CALCOCO3/TAX1BP1 (Chen et al. 2022). NDP52 is a member of the nucleus point family and is distributed in both the cytoplasm and nucleus. The amino end is the SKICH30 domain, the central region is the curly spiral domain, and the carboxyl end is composed of one LIM domain and two zinc finger structures (Morriswood et al. 2007). It is the primary receptor for PINK1/Parkin-mediated mitophagy. The ubiquitin kinase PINK1 recruits NDP52 and Optineurin to mitochondria to directly activate mitophagy independent of Parkin (Michael et al. 2015). In addition, NDP52 is also involved in xenophagy and immune response (Sharma et al. 2018; Leymarie et al. 2017). TAX1BP1 is a selective macro-autophagy/autophagy receptor that plays a central role in host defense against pathogens and regulation of the innate immune system. TAX1BP1 can also perform a variety of auxiliary functions, affecting the biogenesis and maturation of auto-phagosomes (Chen et al. 2022). Other study has shown that TAX1BP1 also plays a role in the negative regulation of cell growth and apoptosis (Verstrepen et al. 2011).

OPTN, like NDP52, it functions as a pink1 downstream protein and contains several structural domains, including two coiled-coil domains, a leucine zipper domain, an LC3-interacting region (LIR), a ubiquitin-binding domain and a zinc finger domain. OPTN-mediated autophagy-dysfunction is closely related to a variety of diseases, such as Neurodegenerative diseases, Neurodegenerative diseases, Cancer, and Nephropathy. Other study has shown the HACE1–optineurin axis promotes tumor suppression in an ubiquitin-dependent fashion (Liu et al. 2014). And OPTN play distinctive roles in different diseases, depending on its primary molecular function in each respective condition (Qiu et al. 2022).

NBR1 was discovered as a selective autophagy receptor due to its interaction with and similarity in domain organization to p62 and direct binding to ATG8 proteins and ubiquitin (Kirkin 2009). NBR1 and p62 share an N-terminal PB1 domain, the ZZ zinc finger domain, LIR motif, and C-terminal UBA domains. In addition, NBR1 contains the four tryptophan domains involved in protein–protein interactions, two coiled-coil domains, and an amphipathic helix domain not found in p62 (Rasmussen et al. 2022). Besides, it also plays an important role in cancer metastasis and immune evasion (Yamamoto et al. 2020; Marsh and Debnath 2020).

In addition to SLRs, some mitochondrial membrane proteins can also bind directly to LC3 without relying on ubiquitin. The common feature of these proteins is the conserved key region, which could directly bind to ATG8/LC3 or other proteins in the family. The following proteins are classified and described according to the location in mitochondria:

### OMM proteins

FUNDC1 is a 155-amino acid macromolecular protein with 3 transmembrane domains, in addition, the N-terminal domain exposed to the cytoplasm contains a domain LIR that interacts with LC3 (Qiu et al. 2022). Under normal circumstances, phosphorylated FUNDC1 is present in the OMM, and when in a hypoxic environment, unc-51-like kinase 1 (ULK1) phosphorylated Ser17 on FUNDC1, and phosphoglycerate mutase family member 5 (PGAM5) dephosphorylates Ser13, thereby promoting FUNDC1 LIR interacts with LC3 to initiate mitophagy occurring. Other study has shown that FUNDC1 is also involved in regulating mitochondrial fission and fusion to help mitochondrial quality control (Chen et al. 2016a, b).

NIX, also known as BNIP3L, is a member of the B cell lymphoma-2 (Bcl-2) family and has 56% homology with BNIP3. It is a mitochondrial autophagy receptor located in the outer membrane of the mitochondria. During the maturation of mammalian erythrocytes, Nix-mediated mitochondrial autophagy plays an important role in the removal of mitochondria (Ashrafi and Schwarz 2013). The related mechanism may be that NIX acts as an autophagy receptor to recruit autophagy-related molecules to initiate autophagy (Zhang and Ney 2009). In addition, NIX is similar to BNIP3 in function, and its C-terminal transmembrane domain induces apoptosis by interacting with Bcl-2 and BCL-XL (Diwan et al. 2007; Zhang and Ney 2009). NIX protein can not only induce apoptosis but also mediate mitochondrial autophagy (Dorn 2010).

BNIP3 and NIX are both members of the Bcl-2 family containing only the BH3 domain subfamily (Lampert et al. 2019). BNIP3 and NIX were found to be a class of pro-apoptotic proteins (Fei et al. 2004), which are highly expressed in cancer cells and cardiomyocytes under hypoxic conditions and are closely related to cell death. Then these two proteins were found that both of them also play an important role in promoting mitophagy of cell survival. BNIP3 induces the release of Beclin-1 by competitively binding Bcl-2 with the autophagy core protein Beclin-1, and then activates autophagy. In addition, there is a conservative LIR that recognizes auto-phagosome LC3 at the N-terminal of BNIP3, which can promote mitophagy. Several studies have shown that elevated mitochondrial ROS increases the expression of BNIP3/NIX to trigger mitophagy (Chourasia et al. 2015; Li et al. 2015; Hu et al. 2016).

Bcl2L13 is also a member of the Bcl-2 protein family and is in the OMM. It has four conserved BH structures and one transmembrane structure of the Bcl-2 family (Meng et al. 2021). Bcl2L13 is a newly reported mitophagy receptor, which is a homologous protein of mitophagy-related gene ATG32, which mediates the clearance of damaged mitochondria (Murakawa et al. 2015.). In addition, like all members of the Bcl-2 protein family, Bcl2L13 has the function of regulating apoptosis (Meng et al. 2021).

FKBP8 is a member of the FK506-binding protein family and the only member of the family with a transmembrane domain, which enables it to be located on the membrane, such as mitochondria and endoplasmic reticulum (Misaka et al. 2018; Shirane et al. 2003). In addition, FKBP8 has four domains: peptidyl-prolyl cis–trans isomerase domain, TPR sequence, calmodulin-binding site and glutamate-rich region. FKBP8 can regulate mitophagy by combining LIR with autophagosome marker protein LC3 (Bhujabal et al. 2017). Different from other autophagy receptors, FKBP8 can transfer from mitochondria to endoplasmic reticulum during mitophagy, to avoid being degraded by auto-phagosomes together with damaged mitochondria. The mechanism of this escape remains to be studied. In addition, FKBP8 can regulate apoptosis through peptidyl-prolyl *cis*–*trans* isomerase domain, TPR sequence, calmodulin-binding site, and apoptosis regulator Bcl-2 (Edlich and Lücke 2011).

AMBRA1 is composed of 1300 amino acids with a molecular weight of about 130 kDa. When mitochondria are damaged, the depolarization of mitochondrial membrane potentials significantly increases the interaction between Parkin and Ambra1. Ambra1 are recruited around the depolarized mitochondria in a Parkin-dependent manner, activating the PI3KIII complex and promoting its clearance through autophagy. In addition, Ambra1-LC3 interaction can transport damaged mitochondria to autophagosomes, which is essential for amplifying Parkin-mediated mitochondrial clearance. Ambra1 can mediate mitophagy in human neuroblastoma SH-SY5Y cells, thus inhibiting oxidative stress and apoptosis induced by 6-hydroxydopamine and rotenone (Di Rita et al. 2018). Many studies have shown that Ambra1-mediated autophagy may be an important mechanism of drug resistance. At the same time, Ambra1 may also play a role in tumor inhibition by regulating other signal pathways (Liu et al. 2017; Sun et al. 2019, 2018).

NLRX1 (nucleotide-binding domain and leucine-rich-repeat-containing protein X1) belongs to the NLR family of intracellular sensors that regulate major cellular pathways including cell death and inflammation (Imbeault et al. 2014). In addition, study has shown that it is also related to cellar apoptosis (Soares et al. 2014).

### IMM proteins

PHB2 belongs to the anti-proliferative protein family PHBs, which mainly encodes two protein members, PHB1 and PHB2 in the human genome. PHB1 and PHB2 can be combined as heterodimers to form a rosette palisade protein complex anchored in the IMM, which is involved in maintaining the stability of mitochondrial structure, regulating mitochondrial dynamics and mitochondrial crest morphology, regulating mitochondrial differentiation and development, and maintaining the stability of mitochondrial genome (Merkwirth and Langer 2009). PHB2 contains transmembrane domain (amino acid 1–36), central prohibitin domain (amino acid 36–201) and overlapping coiled helix domain (amino acid 188–264) for mitochondrial localization. In addition, PHB2 also contains estrogen receptor binding domain. It is mainly located in mitochondria, but also in cytoplasm, nucleus and plasma membrane (Kuramori et al. 2009). Prohibitin-2 can stabilize PINK1 on the OMM to mediate the PINK1-parkin pathway and promote mitophagy (Yan et al. 2020). When the OMM is damaged and the IMM is exposed, it can directly bind to LC3 to assist in the timely phagocytosis and degradation of damaged mitochondria by lysosomes (Wei et al. 2017).

Cardiolipin (CL), is a kind of mitochondrial-specific phospholipid, which is mainly distributed on the IMM. It is composed of two phosphoric acid molecules and three glycerol molecules, and then connected with four fatty acid molecules. CL can produce negative curvature elastic stress in lipid bilayer membrane, so it plays a key role in maintaining a certain curvature of mitochondrial inner membrane crest (Ikon and Ryan 2017). Chu et al. confirmed that CL can be transported from the IMM to the OMM after mitochondrial injury, which directly interacts with LC3 and mediates mitophagy (deArriba et al. 2013; Fernandez et al. 2002; Chu et al. 2013). In addition, cardiolipin–cytochrome C complex can also regulate apoptosis (McMillin and Dowhan 2002).

## Mitophagy-related signaling pathways (shown in Fig. 2)

**Fig. 2:**
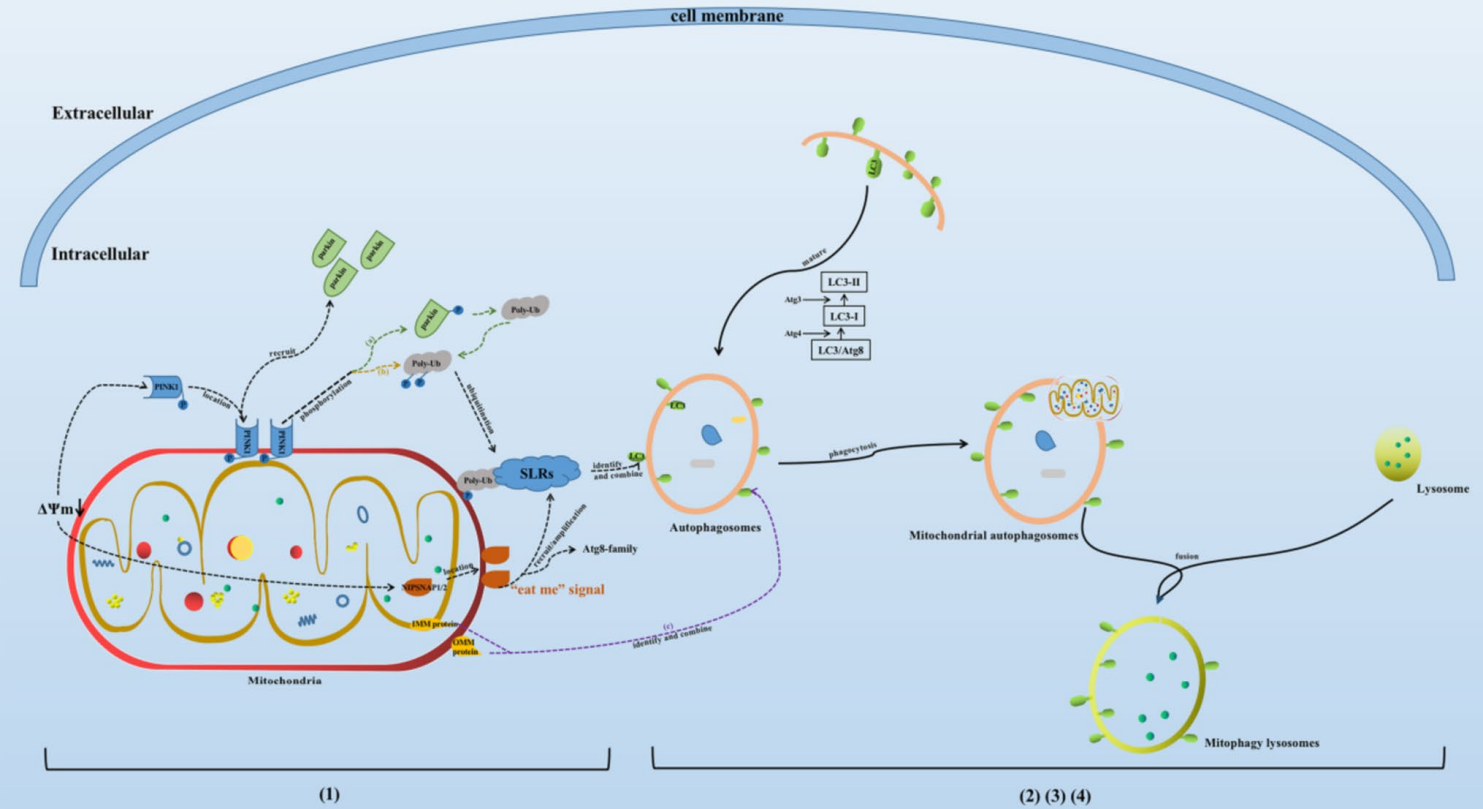
Process and related pathways of mitophagy. Note: (1) mitochondrial damage leads to mitochondrial depolarization and induces the activation of mitophagy-related proteins; (2) autophagosomes wrap damaged mitochondria to form mitochondrial autophagosomes; (3) lysosome fusion to form mitochondrial autophagy lysosomes; (4) degradation and recycling of related substances; (a) PINK1-parkin pathway; (b) Parkin-independent pathway; (c) direct interaction of LC3 with IMM/OMM proteins

### PINK1-parkin pathway

PINK1-parkin is currently one of the most classic signaling pathways of mitophagy. PINK1 is a 581 amino acid protein with serine/threonine protein kinase activity, consisting of an N-terminal mitochondrial targeting motif that contains a transmembrane domain (110 amino acids long), a highly conserved kinase domain with three insertions in the N lobe, and a C-terminal autoregulatory sequence (Kumar et al. 2017). PINK1 is synthesized in the cytoplasm and is very low in normal mitochondria, but it could accumulate in the OMM when mitochondria are under depolarizing state, which could act as molecular receptors for damaged mitochondria. Then it phosphorylates Parkin and ubiquitin to recruit them to the damaged mitochondria, leading to the ubiquitination of mitochondrial proteins and the initiation of mitophagy. Parkin is a downstream protein of PINK1, an E3 ubiquitin ligase composed of 465 amino acids encoded by the PRKN gene, which mainly mediates substrate ubiquitination, regulates protein degradation and signal transduction. PINK1 acts as a key sensor of mitochondrial damage, whereas Parkin amplifies this damage signal by facilitating the formation of ubiquitin chains, which recruit more Parkin to the damaged mitochondria. Phosphorylated parkin cascades amplify ubiquitination signals and initiate mitophagy. Lin et al. found that PINK1-parkin pathway of mitophagy protects cells via decreasing mitochondrial ROS (Lin et al. 2019).

Additionally, parkin-dependent ubiquitination of OMM proteins is necessary for mitophagy, it probably acts as a priming event that allows the OMM localized NIPSNAP1 and NIPSNAP2 to recruit the autophagy receptors. Several studies have shown that NIPSNAP1 and NIPSNAP2 will locate in the OMM when mitochondria are depolarized by external stimulation, sending out “eat me” signals to recruit ATG8-family proteins and ubiquitin-dependent SLRs to mediate more powerful mitophagy (Abudu et al. 2019; Princely Abudu et al. 2019).

### Parkin-independent pathway

The depolarization of the mitochondrial membrane makes PINK1 stable in the OMM. PINK1 can directly exert its own kinase activity without parkin, phosphorylate and activate ubiquitin proteins, and then ubiquitinate the proteins related to autophagy. Polyubiquitin chain plays the role of promoting mitophagy (Ordureau et al. 2014).

### Other pathway

In addition, there are some pathways that are independent of parkin and ubiquitin, and directly mediate the process of mitophagy through mitochondrial membrane proteins. The OMM and IMM proteins mentioned in “OMM proteins” and “IMM proteins” above can start the process of mitophagy binding to LC3 on autophagy lysosomes.

## Relationship between mitophagy and radiosensitivity of cancer cells

According to the current research on mitophagy in cellular radiosensitivity, as shown in Fig. 1, there are mainly the following two perspectives:

On the one hand, mitophagy can enhance the radio-resistance of cancer cells. That is, mitophagy play a protective role in irradiated tumor cells. The existence of mitophagy can identify and clear the damaged mitochondria early, the decrease of the number of mitochondria inhibits the process of oxidative phosphorylation and compensatively induces glycolysis, and glycolysis increases the contents of lactic acid, pyruvate, and ketone bodies in cells. These metabolites are secreted by tumor matrix and reused by cancer cells to meet their metabolic needs and maintain intracellular homeostasis. Besides, the rapid renewal of mitochondria leads to the presence of a large number of newly generated mitochondria (Wu et al. 2022). Several research results support the above point of view. Chen et al. demonstrated through in vivo and in vitro experiments that the LACTB2 protein rendered nasopharyngeal carcinoma resistant to radiation therapy, and PINK1/Parkin-mediated mitophagy induced a healthier mitochondrial network and contributed to radio-resistance of nasopharyngeal carcinoma (Chen et al. 2021). Yang et al. have shown that mitophagy can resist oxidative stress caused by IR, and inhibition of mitophagy can increase the accumulation of ROS and induce cancer cell death (Yang et al. 2021). Wang et al. found that protein disulfide isomerase can inhibit radiotherapy-induced cell death by regulating mitophagy signaling, increasing cellular radio-resistance. Cancer stem cells population has higher mitophagy level, which could promote tumorigenesis and cell survival in various tumor types by allowing the removal of abnormal mitochondria (Baghban et al. 2020; Takeda et al. 2019). Fan et al. also proved that early oxidative stress enhanced mitophagy to protect cells, but mitophagy was reduced and apoptosis was increased once the cells had irreversible damage after long-term oxidative exposure (Fan et al. 2019). Wu et al. found that the level of mitophagy was elevated in radio-resistant A549R cells, and inhibition of mitophagy can increase the radiosensitivity of A549R cells (Wu et al. 2022), they believe that it is related to ROS and DNA damage.

On the other hand, increasing radiosensitivity of cancer cells. This view is mainly related to intracellular ROS and DNA damage. DNA damage is closely related to tumor occurrence and development. Ren et al. found that mitophagy, as an upstream signal, increases ionizing radiation-induced DNA damage by downregulating or overexpressing key mitophagy proteins Parkin and BNIP3 (Ren et al. 2023). H_2_O in irradiated cells can be dissociated to ROS including H_2_O_2_ and ${\text{O}}_{2}^{ \cdot - }$, then a cascade reaction will tend to occur. Furthermore, mitochondria are also the sources of ROS. Strong external stimulation causes excessive mitochondrial damage, serious imbalance of cellular oxidative phosphorylation, excessive accumulation of ROS, inducing excess mitochondria are removed and causing cell death. Yu and Chen et al. irradiated Hela and MCF-7 cells and found that radiation can induce autophagy, resulting in increased intracellular ROS levels and increased mitochondrial damage, mitophagy here increases the sensitivity of cells to IR (Yu et al. 2021; Chen et al. 2016a, b). In addition, insufficient mitophagy can lead to the accumulation of harmful substances and apoptosis or death of cells.

## Conclusion

This review summarized the receptors and pathways related to mitophagy, and mainly discussed the relationship between mitophagy and radiosensitivity of cancer cells around mitochondria and IR. In summary, IR as an external stimulus can affect the occurrence and activity of mitophagy of cancer cells. Mitophagy plays a “Double-edged sword” role in the radiosensitivity of cancer cells, and the time–effect relationship and dose–effect relationship need to be further explored.

## Data Availability

The datasets generated during and/or analysed during the current study are available in the PubMed: https://pubmed.ncbi.nlm.nih.gov/.

## Abbreviations

ROS: Reactive oxygen species
IR: Ionizing radiation
ATP: Adenosine triphosphate
OMM: Outer mitochondrial membrane
IMM: Inner mitochondrial membrane
TCA: Tricarboxylic acid cycle
NOX: NADPH oxidase
RNS: Reactive nitrogen species
ΔΨm: Mitochondrial membrane potential
mtDNA: Mitochondrial DNA
rRNAs: Ribosomal RNAs
tRNAs: Transfer RNAs
CaMK: Calcium ion/calmodulin kinase
FC: Flow cytometry
LC3: Light chain 3
ATG: Autophagy-related protein
SLRs: SQSTM1-like receptors
LIR: LC3-interacting region
ULK1: Unc-51-like kinase 1
PGAM5: Phosphoglycerate mutase family member 5
Bcl-2: B-cell lymphoma-2
CL: Cardiolipin
